# Supplementary material for: Geometrical congruence, greedy navigability and myopic transfer in complex networks and brain connectomes
Source: Nat Commun. 2022 Nov 27;13:7308. doi: 10.1038/s41467-022-34634-6 (PMC9701786; doi:10.1038/s41467-022-34634-6)
Supplement: Supplementary file 1 — Supplementary Information [file 41467_2022_34634_MOESM1_ESM.pdf]

Geometrical congruence, greedy navigability and myopic transfer in complex networks and brain connectomes

Carlo Vittorio Cannistraci[1,2,3,4,5,6,*] and Alessandro Muscoloni[1,5]

[1] Center for Complex Network Intelligence (CCNI), Tsinghua Laboratory of Brain and Intelligence (THBI), Tsinghua University, 160 Chengfu Rd., SanCaiTang Building, Haidian District, 100084, Beijing, China

[2] Department of Computer Science, Tsinghua University, Beijing, China

[3] Department of Biomedical Engineering, Tsinghua University, Beijing, China

[4] Department of Physics, Technische Universität Dresden

[5] Biomedical Cybernetics Group, Biotechnology Center (BIOTEC), Center for Molecular and Cellular Bioengineering (CMCB), Tatzberg 47/49, 01307 Dresden, Germany

[6] Center for Systems Biology Dresden (CSBD), Pfotenhauerstr. 108, 01307, Dresden, Germany

* Corresponding author: Carlo Vittorio Cannistraci (kalokagathos.agon@gmail.com)

Supplementary Information

Suppl. Note 1. Previous measures for evaluation of greedy routing navigability and their limitations

In 2010, Krioukov et al.[1] suggested that the underlying geometry of hyperbolic networks generates a hierarchical and clustered topology that enables 'myopic transfer' (greedy forwarding) to form greedy routing paths (GRP) that follows the geodesics in the hyperbolic space. To arrive to this conclusion, Krioukov et al.[1] propose some greedy routing navigability measures that can evaluate the quality of greedy routing paths in (hyperbolic) networks, and therefore can help to determine whether the greedy navigation is efficient or not in such networks. In order to introduce these measures, we have to offer more formal details on the greedy routing navigability process. The 'myopic transfer' that creates a greedy routing path between a source node *i* and a destination node *j* works as follow. A packet is sent across the network from the

source *i* to the destination *j*. Every node knows only the geometrical coordinates (address) of its neighbours and the ones of the destination *j*, which is written in the packet. At each step (hop) the packet is transferred from the current node to its neighbour closest to the destination, meaning at the lowest geometrical distance (hyperbolic distance, in the study of Krioukov et al.[1]). Since each node knows only its own address, the addresses of its neighbours, and the destination address of the packet, no node has global knowledge of the network structure. The transfer of the packet stops when:

(i) it successfully reaches the destination node *j*. In this case, both the number of GRP hops (hGRP) and the projection of the greedy routing path (pGRP) in the geometrical space are interesting measures to consider.

(ii) it unsuccessfully gets stuck before to arrive to destination. This happen when the neighbour closer to destination is the same node from which the packet has been received at the previous step. Since a loop has been generated, the greedy routing is unsuccessful (pGRP is set to infinite). There is also a variation of this rule according to which the algorithm drops the packet if the current node is a local minimum, meaning that it does not have any neighbour closer to the destination than itself. However, Krioukov et al.[1] demonstrated that the two variations (named respectively modified (MGR) and original (OGR) greedy routing) display a similar behaviour in its computational experiments, therefore we will consider only the MGR version that is the most adopted one in literature.

The measures proposed by Krioukov et al.[1] to estimate the GR navigability performance are the following:

(1) the success GRP ratio, which is the percentage of successful GRPs, computed as the proportion of GRPs that reach their destinations on all nonadjacent node pairs.

(2) the average hop length of successful GRPs.

(3) The standard hop stretches (indicated with the acronym S1), defined as the ratio between the hop lengths of successful GRPs and the hope length of the associated topological shortest paths between the same pair of nodes in the graph.

All these three measures are merely topological, and they do not consider the underlying geometry. Differently, the measures described below are *hyperbolic stretches* and take into consideration also the underlying hyperbolic geometrical space as a reference.

(4) the average hyperbolic stretch (indicated with the acronym S2) of the successful GRPs projections (pGRP) with respect to a nongreedy geometrical reference that is the GEO. Note that only node pairs for which a successful GRP exists are considered.

(5) the average hyperbolic stretch (indicated with the acronym S3) of the successful GSPs with respect to a nongreedy geometrical reference that is the GEO. Note that only node pairs for which a successful GRP exists are considered.

(6-7) Krioukov et al.[1] considered for technical reasons also the maximum instead of the average S2 and S3 stretches, but we omit to discuss these two measures because of less relevance and less usage in literature.

We clarify that the model of hyperbolic networks of Krioukov et al.[1] as the following parameters: node size; average degree; power-law exponent γ (generally between 2 and 3, under value 3 the network is considered to acquire a marked heterogeneous and hierarchical structure); temperature T (generally between 0 and 1, the closer to 0 the more the network is clustered, the closer to 1 the more is random).

The hyperbolic stretch measures above do not account for all the network topology but only for the one visited by successful GRPs, indeed they are defined only for the nonadjacent nodes pairs that are connected by a successful GRP. Therefore, these two measures are well-defined to evaluate greedy navigability only when the success GRP ratio is high. This is actually happening only in a specific case, when the temperature T = 0 (Fig. 9, first plot on the top, in Krioukov et al. [1]) in the hyperbolic model adopted by Krioukov et al.[1]. But, when the temperature grows, the percentage of unsuccessful GRPs increases significantly (Fig. 11, first plot on the top, in Krioukov et al. [1]). Unlikely, many real networks do not fit with the ideal scenario of T = 0, and therefore there are a relevant amount of unsuccessful GRPs. Hence, there is clear need to invent new measures that evaluate greedy navigability efficiency holistically, taking into account both successful and unsuccessful paths. Nevertheless, Krioukov et al.[1] in their article claim (text is reported verbatim): << The lower these two stretches [S2 and S3], the closer the greedy and

shortest paths stay to the hyperbolic geodesics, and the more congruent we say the network topology is with the underlying geometry >>. This statement to a certain extent can be also considered misleading, because, as said above, many GRPs can be unsuccessful[1] and a consistent part of nonadjacent node pairs can be neglected in the computation of S2 and S3 measures, therefore these two measures are not apt to evaluate congruence. Indeed, S2 and S3 assess how on average successful GRPs (S2) and the 'associated GSPs' (S3) are geometrically stretched in comparison to a non-greedy geometrical reference such as the GEO. On the other side, to be fair, Krioukov et al.[1] never claimed in their study that S2 and S3 are designed to be measures of network congruence, but they implied that, to a certain extent and for what regards only the part of topology that is explored by successful GRPs, S2 and S3 can offer approximated information respectively on the congruence of the GRPs (S2) and 'the associated GSPs' (S3) with the underling geometry. Yet, a well-posed measure that exactly evaluates the congruence of a network topology with the underlying geometry should summarize the deviation of the projections of all the topological shortest paths (pTSPs) with respect to a geometrical reference. Boguñá, Krioukov and colleagues[2] agree with this need to consider all shortest paths to evaluate congruence, indeed, in a recent review on network geometry[2], they stated (text is reported verbatim): << Geometric navigation is effective only when the network topology is congruent with the underlying latent geometry so that following geodesic paths in the latent space is equivalent to navigating through topological shortest paths. >>. Then, in the same review article they noted, by referring to the pre-print version of this article, that a valid definition and measure for congruency could be also by taking into account all topological shortest paths[2]. However, this was not possible to be achieved in 2010 in the study of Krioukov et al.[1], because it was not available an efficient algorithm to detect, enumerate and measure the projections of all the possible shortest paths between nonadjacent pairs of nodes in a network, which is a demanding computational problem. In our study we present an efficient algorithm to address this computational problem in large networks, therefore we can propose a measure of congruence that is general (not only for hyperbolic networks) and exact (because it considers all possible shortest paths).

Before to proceed with discussing our innovations (in the section of the main article: "The geometrical congruence problem"), we should also comment the finding that Krioukov et al.[1] claimed on congruence in hyperbolic networks using the S2 and S3 stretch measures which, we stress, can suggest approximated knowledge on the congruence but are not defined to be, and they are not, an exact measure of congruence.

Krioukov et al. arrived at the conclusion that (text is reported verbatim): << Since the GF paths, also the shortest paths in the network, follow the shortest geodesic paths in the hyperbolic space, the resulting hyperbolic stretch is small. >>. Then, on the basis of this comment that, we emphasize, has its key point in the fact that the "hyperbolic stretch is small", they concluded that hyperbolic networks are maximally efficient for geometric navigation[1,2]. The main reason behind this phenomenon according to Krioukov and colleagues is that, as soon as $\gamma < 3$, there is the existence of greedy and shortest paths close (congruent) to the corresponding geodesics in the underlying hyperbolic geometry (Fig. 13a of Krioukov et al.[1]) for any pair of nodes in hyperbolic networks[1–3]. Therefore, navigation in hyperbolic networks with $\gamma < 3$ is believed to always find these ultrashort paths [1–3].

But, are the hyperbolic stretches reported in the study of Krioukov et al. small enough to claim that the portion of successful GRPs follow and therefore are congruent with the geodesics? For hyperbolic networks generated at $T = 0$ and $\gamma = 2$ (Fig. 9, third plot from the top[1]), Krioukov et al.[1] report hyperbolic stretches (S2 and S3) of around 1.5, meaning that the geometrical length of the GRP (or associated GSP) is on average 50% larger than the one of the GEO. For hyperbolic networks generated at $T = 0$ and $\gamma = 2.5$, the hyperbolic stretches are around 2, meaning that the geometrical length of the GRP (or associated GSP) is on average 100% larger than the one of the GEO. For hyperbolic networks generated at $T = 1$ and $\gamma = 2.1$, they report hyperbolic stretches of around 2, meaning that the geometrical length of the GRP (or associated GSP) is on average 100% larger than the one of the GEO. The first consideration is that deviations of 50% and 100% are in general too large to claim that two paths are congruent, indeed in the Fig. 13a of Krioukov et al.[1] appears visually evident that the GRPs largely differs and do not follow closely the GEO. More precisely, Fig. 13a of Krioukov et al.[1] shows a visual example of the deviation of two different GRPs (which are also GSP: S2 = S3, which occurs frequently when the T=0) from GEO. The two

GRPs have the same source node at the top but they progress towards two different destinations. The hyperbolic stretches of the left and right paths are 1.51 and 1.68 respectively, which means in general more that 50% error in comparison to the GEO. Indeed, there is not geometrical congruence between the trajectories and the GRPs (or associated GSPs) and GEOs curves. However, there is a second consideration that is even more convincing on the fact that these measures S2 and S3 can be misleading. In the hyperbolic network model used by Krioukov et al.[1], the temperature T is a parameter the control the clustering of the network. $T = 0$ means very high clustering and therefore preservation of the hyperbolic geometry, $T = 1$ means high random connectivity, which means using the same expression of Krioukov et al.[1] (text is reported verbatim): << As clustering weakens, not only the path diversity in the network decreases, but also the network metric structure deteriorates since the edge existence probability (41) depends less and less on the hyperbolic distance between nodes. In the extreme case of classical random graphs, for example, the connection probability does not depend on this distance at all. As a result, the congruency between network topology and underlying geometry evaporates >>. Therefore, in the results of Krioukov et al.[1], a hyperbolic network at $T = 0$ and $\gamma = 2.5$ - which is for sure respecting the underlying geometry because the temperature is zero - and a network at $T = 1$ and $\gamma = 2.1$ - generated with the same model but in a way to have a random connectivity that negligibly respects the underlying geometry - have the same S2 and S3 equal to around 2 which means that in both scenarios the GRPs (or GSPs) are on average 100% larger than GEO. This is clearly a logical absurdity and, since we are confident to say that an error of 100% is large enough to reject possibility of high congruence between the topology and the geometry, we should equally reject the hypothesis that hyperbolic networks are congruent with the underlying geometry triggering maximal navigability. This analysis suggests that the statement of Krioukov et al., on the fact that navigation in hyperbolic networks with $\gamma < 3$ is maximally efficient [1–3] because GRPs follow closely the GEOs, is not convincing. Indeed, what is missing in the formula of S2 and S3 to be a more robust estimator of navigability is to account also the GRP success ratio that is close to 1 for $T = 0$ (Fig. 9, first plot on top of Krioukov et al.[1]) and $\gamma = 2.5$ and is close to 0.5 (half GRP success) for $T = 1$ (Fig. 11, first plot on top of Krioukov et al.[1]). Adjusting S2 and S3 to take into consideration also the number of unsuccessful GRPs would make S2 and S3 at $T = 1$

much larger and therefore would clarify that the navigability at T = 1 is less efficient than at T = 0. But this adjustment would not still adapt S2 and S3 to be measure of congruence because they are based on greedy paths that can fail not saying anything on the congruence but only on the navigability. For assessing the congruence, it is necessary to take into account, for each nonadjacent node pair, all the shortest paths in the network, that is what we achieve in this study.

Suppl. Note 2. Pseudocode to compute the $\overline{pTSP}$ between all node pairs.

INPUT
N – number of nodes
A – adjacency list, containing for each node the list of neighbours;
A[1] is the list of neighbours of node 1, A[2] the same for node 2, and so on for N nodes.
G – NxN matrix of geodesics between all node pairs.
T – NxN matrix of topological shortest paths between all node pairs.

OUTPUT
P = NxN matrix of $\overline{pTSP}$ between all node pairs.

```
function P = compute_pTSP(A, T, S, order)
# compute for each node the mean of the topological shortest paths to all other nodes
Tmean = numerical vector of N elements, initialized to zeros          O(N)
for s in [1…N]                                                        O(N)
    for t in [s+1…N]                                                  O(N)
        Tmean[s] = Tmean[s] + T[s,t]                                  O(1)
        Tmean[t] = Tmean[t] + T[s,t]                                  O(1)
    Tmean[s] = Tmean[s] / (N-1)                                       O(1)
# sort nodes by decreasing mean of the topological shortest paths
order = numerical vector of N elements, initialized to zeros          O(N)
order = get_sort_indexes(Tmean, 'decreasing')                         O(NlogN)
# the hypothetical function get_sort_indexes sorts the elements of Tmean
# by decreasing order and returns the indexes of the sorted elements
# compute L, which indicates for each node the maximum path length to evaluate
L = numerical vector of N elements, initialized to zeros              O(N)
mask = logical vector of N elements, initialized to false             O(N)
for i in [1…N]                                                        O(N)
    s = order[i]                                                      O(1)
    for t in [1…N]                                                    O(N)
        if (mask[t]==false) & (T[s,t]>L[s])                           O(1)
            L[s] = T[s,t]                                             O(1)
```

```
        mask[s] = true                                                          O(1)

# main section
P = numerical matrix NxN, initialized to zeros                                  O(N^2)
for i in [1...N]   # can be run in parallel                                     O(N)
        lt = 0                                                                  O(1)
        lg = 0                                                                  O(1)
        Pcount = numerical vector of N elements, initialized to zeros           O(N)
        inpath = logical vector of N elements, initialized to false             O(N)
        s = order[i]                                                            O(1)
        inpath[s] = true                                                        O(1)

        # start recursive computation for each neighbor of s
        for t in A[s]                                                           O(k_s)
                compute_pTSP_rec(N, A, G, T, L, P, Pcount, inpath, s, t, lt, lg, G[s,t])

        # update P
        for t in [1...N]                                                        O(N)
                if (L[s]>L[t]) | ((L[s]==L[t]) & (s<t))                         O(1)
                        P[s,t] = P[s,t] / Pcount[t]                             O(1)
                        P[t,s] = P[s,t]                                         O(1)

# Note: the matrices and vectors variables A, G, T, L, P, Pcount, inpath
# are passed by reference to the function compute_pTSP_rec
# therefore they are shared and not copied

function compute_pTSP_rec(N, A, G, T, L, P, Pcount, inpath, s, t, lt, lg, g)

inpath[t] = true        # add visited node to current path                      O(1)
lt = lt + 1             # topological length of current path                    O(1)
lg = lg + g             # geometrical length of current path                    O(1)

# update P and Pcount if needed
if (lt==T[s,t]) & ((L[s]>L[t]) || ((L[s]==L[t]) & (s<t)))                       O(1)
        Pcount[t] = Pcount[t] + 1                                               O(1)
        P[s,t] = P[s,t] + lg                                                    O(1)

# if maximum path length to evaluate not reached, continue recursion
if lt < L[s]                                                                    O(1)
        for u in A[t]                                                           O(k_t)
                if inpath[u]==false                                             O(1)
                        compute_pTSP_rec(N, A, G, T, L, P, Pcount, inpath, s, u, lt, lg, G[t,u])

inpath[t] = false       # remove visited node from current path                 O(1)
```

Suppl. Note 3. Space and time complexity

In this section we discuss space and time complexity of the algorithm for computing the $\overline{pTSP}$ between all node pairs, as presented in Suppl. Algorithm 1.

Let's start with the space complexity and analyze the variables in the pseudocode of the algorithm. The variables G, T and P are matrices of size $N \cdot N$, therefore $O(N^2)$, where N is the number of nodes. The variable A is an adjacency list, whose space complexity is $O(E)$, where E is the number of edges, which in the worst case of a fully connected network becomes $O(N^2)$. The variables Tmean, order, L, mask, Pcount and inpath are vectors of size N, therefore $O(N)$. Note that in case of parallel computation the variables Pcount and inpath are allocated for each parallel thread, for a total of $p \cdot N$ elements, where p is the number of parallel threads. However, for networks of large size we have $p \ll N$ (for example in our largest simulations we have $p = 128$ and $N = 100000$) and p is usually a constant factor within a computational environment, therefore we can approximate to $O(N)$. All the other variables in the pseudocode have $O(1)$ space complexity. Therefore, the algorithm has $O(N^2)$ space complexity, which scales well for networks of large size.

Let's proceed now with the time complexity analysis. The cost of individual operations is annotated in Suppl. Algorithm 1. In lines 4-9 we compute for each node the mean of the topological shortest paths to all other nodes, which requires $\frac{N(N-1)}{2}$ loop iterations, therefore the overall cost is $O(N^2)$. In lines 12-13 we sort the nodes by decreasing mean of the topological shortest paths, which can be performed in $O(NlogN)$. In lines 18-25 we compute for each node the maximum path length to evaluate (recursion depth), which requires N^2 loop iterations, therefore the overall cost is $O(N^2)$. Looking at the main section of the code, the initializations at lines 28, 32, 33 require overall $O(N^2)$. Lines 42-45, where the output variable P is updated, also require N^2 loop iterations and an overall cost of $O(N^2)$. Until now, all these processing steps have a time complexity of $O(N^2)$.

Finally, we will focus to analyze the recursive part of the algorithm. For each source node (line 29), for each of its neighbours (line 38), we start a recursive visit (line 39) up to a pre-computed recursion depth associated to the source node. Although different nodes visited during the recursion might have a different number of neighbours to recursively visit, in order to make the

analysis tractable we will consider for each node a number of neighbours equal to the average node degree k. Under this assumption, the source node i will traverse a number of paths approximated by k^{L_i}, where L_i is the recursion depth of node i. Adding up the contributions of all source nodes, the time complexity of the recursive part of the algorithm is:

$$T = O\left(\sum_{i=1}^{N} k^{L_i}\right)$$

The recursion depths can range between a minimum of 2 up to a maximum equal to the diameter d of the network. Let's define M_l as the proportion of nodes with a certain recursion depth l, then we consider $M_2 \cdot N$ nodes with recursion depth $L = 2$, $M_3 \cdot N$ nodes with recursion depth $L = 3$, and so on until $M_d \cdot N$ nodes with recursion depth $L = d$. We can decompose the time complexity as:

$$T = O\left(N \cdot \sum_{l=2}^{d} M_l \cdot k^l\right) = O(N \cdot (M_2 \cdot k^2 + M_3 \cdot k^3 + \cdots + M_d \cdot k^d))$$

The analysis of this formulation is not trivial, since it depends on the distribution of the recursion depths, which in turn are affected by different topological features of the network. One of such features is certainly the average node degree (or similarly the density or the number of edges). The higher the average node degree, the shorter will be the topological shortest paths, the lower will be the recursion depths. In case of networks with a power-law degree distribution, such as the nPSO networks analyzed, the power-law exponent γ also affects the recursion depths distribution. Indeed, the lower the γ, the higher the presence of hubs connecting most of the network, making the topological shortest paths and in turn the recursion depths shorter. We analyzed this trend with a quantitative example in Fig. 5c,e,g, which is discussed in the Results section.

Finally, we comment the impact of the last term of the decomposed formulation, $M_d \cdot k^d$, in case of small-world and ultra-small-world networks. For small-world networks[4], we can consider the diameter $d = logN$ and write the last term as $M_d \cdot k^d = M_d \cdot k^{logN} = M_d \cdot N^{logk}$. For ultra-small-world networks[4], we can consider the diameter $d = loglogN$ and we can write the last term as $M_d \cdot k^d = M_d \cdot k^{loglogN} = M_d \cdot (logN)^{logk}$. From the quantitative examples in Fig. 5, we can see that, both for our algorithm and brute-force, the factor M_d (proportion of nodes with

recursion depth equal to the diameter) is generally negligible (M_d ≈ constant). We could therefore approximate the last term of the time complexity for the small-world (for instance γ = 3) and ultra-small-world (for instance γ = 2) cases respectively as N^{logk} and $(logN)^{logk}$. We clarify that although this is not necessarily the dominant term in the time complexity formulation, it has high impact on the running time, and this might explain why there is a significant running time reduction between the networks at γ = 3 and γ = 2.

Suppl. Note 4. Running time estimation

In order to estimate the computational running time, we generated nPSO networks with parameters N = [100, 1000, 10000, 100000], $\bar{d}$ = 12, T = 0.1, γ = [2, 2.25, 2.5, 2.75, 3] and C = 4. For each combination of parameters, 10 networks have been generated. For each network we ran the algorithm to compute the following measures: GC($\overline{pTSP}$, GEO), GC($\overline{pTSP}$, GSP), GRE(pGRP, GEO), GRE(pGRP, GSP).

The computation was executed exploiting several server nodes of the High-Performance Computing (HPC) cluster of ZIH, TU Dresden, each with 512 GB RAM, 2x AMD EPYC CPU 7702 @ 2.0GHz (2x 64 cores). Every network was run independently on a separate machine, therefore the running time measurements are for each single network on a single machine, using 128 parallel threads.

In Fig. 5b, four different estimated running times are reported: our optimized algorithm with parallel computation ($T^{o,p}$), our optimized algorithm with serial computation ($T^{o,s}$), brute-force with parallel computation ($T^{b,p}$), brute-force with serial computation ($T^{b,s}$). The running time that has been actually measured in our simulations is $T^{o,p}$, whereas the other estimated times ($T^{o,s}$, $T^{o,p}$, $T^{b,p}$) have been mathematically derived.

We can decompose the time $T^{o,p}$ as follows:

$$T^{o,p} = T^{o,p}_{pTSP} + T^{p}_{pGRP} + T_{other}$$

where: $T^{o,p}_{pTSP}$ is the time to compute the $\overline{pTSP}$ between all node pairs, using our algorithm and parallel computation; T^{p}_{pGRP} is the time to compute the pGRP between all node pairs, using parallel computation; T_{other} includes all the remaining serial computation, such as the computation of TSP, GSP, GEO, the measures GC and GRE.

Considering c parallel cores (in our setting c = 128) and assuming an equal repartition of the computational load over the cores, we can obtain an estimation of $T^{o,s}$ as:

$$T^{o,s} = \left(T_{pTSP}^{o,p} + T_{pGRP}^{p}\right) \cdot c + T_{other}$$

While running our algorithm we also recorded partial information useful to reconstruct an estimation of the running time for the brute-force variant. The only difference is in the computation of all the topological shortest paths between all node pairs, needed for the measure $\overline{pTSP}$. In particular, as explained in the Methods section, starting from each source node a recursive visit to the neighbours is performed until reaching a certain recursion depth. In the brute-force variant, for each source node i the recursion depth L_i^b is set to its maximum topological shortest path, while in our algorithm the recursion depth L_i^o is optimized in order to avoid redundant computation. Therefore, while running our algorithm on a given network, for each source node i we stored the associated recursion depth L_i^o and the running time t_i^o required for that node to perform a recursion up to that depth. Then, for the brute-force variant, for each source node i we computed the associated recursion depth L_i^b as its maximum topological shortest path, and we assigned a running time t_i^b equal to the mean running time required to reach the same depth in our algorithm:

$$t_i^b = \underset{\forall j:\, L_j^o \equiv L_i^b}{\text{mean}}\, t_j^o$$

Indeed, the time to reach a certain recursion depth does not differ between our algorithm and brute-force, what changes is that brute-force requires to reach longer depths on average.

We can notice that the following equation is valid:

$$T_{pTSP}^{o,s} = T_{pTSP}^{o,p} \cdot c = \sum_{i=1}^{N} t_i^o$$

Therefore, we can rewrite the time $T^{o,s}$ as:

$$T^{o,s} = \left(T_{pTSP}^{o,p} + T_{pGRP}^{p}\right) \cdot c + T_{other} = \sum_{i=1}^{N} t_i^o + T_{pGRP}^{p} \cdot c + T_{other}$$

Finally, we can estimate the times $T^{b,s}$ and $T^{b,p}$ as:

$$T^{b,s} = \sum_{i=1}^{N} t_i^b + T_{pGRP}^{p} \cdot c + T_{other}$$

$$T^{b,p} = \frac{\sum_{i=1}^{N} t_i^b}{c} + T_{pGRP}^{p} + T_{other}$$

In Fig. 5c,e,g, on top of the barplots, we report the mean running time required to reach each recursion depth. In practice, for each recursion depth l, we compute:

$$\overline{T}_l = \underset{\forall i:\, L_i^o \equiv l}{\text{mean}}\, t_i^o$$

In Fig. 5d,f,h we report the running time that would be required to compute the recursive section of the algorithm for each recursion depth and without considering parallel computation, both for our optimized algorithm and brute-force. In practice, for each recursion depth l, we compute:

$$T_l^o = \sum_{\forall i:\, L_i^o \equiv l} t_i^o$$

and

$$T_l^b = \sum_{\forall i:\, L_i^b \equiv l} t_i^b$$

Supplementary References

1. Krioukov, D., Papadopoulos, F., Kitsak, M., Vahdat, A. & Boguñá, M. Hyperbolic geometry of complex networks. *Physical Review E - Statistical, Nonlinear, and Soft Matter Physics* **82**, 036106 (2010).
2. Boguñá, M. *et al.* Network geometry. *Nature Reviews Physics 2021 3:2* **3**, 114–135 (2021).
3. Boguñá, M. & Krioukov, D. Navigating ultrasmall worlds in ultrashort time. *Physical Review Letters* **102**, 058701 (2009).
4. Cohen, R. & Havlin, S. Scale-Free Networks Are Ultrasmall. *Phys. Rev. Lett.* **90**, 58701 (2003).

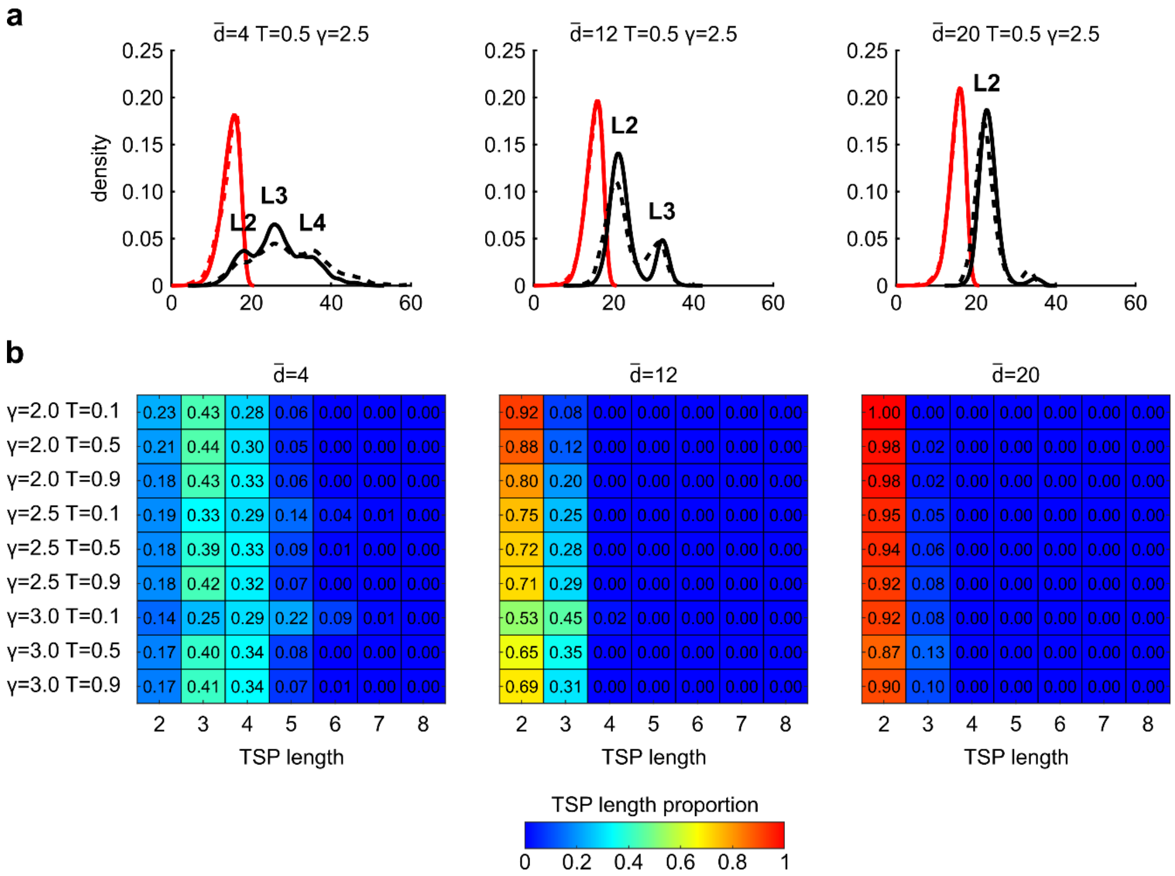

Suppl. Figure 1. TSP length distribution in hyperbolic networks.

(a) The panel is equivalent to panel (a) of Fig. 2, showing the comparison of GEO and $\overline{pTSP}$ distributions in nPSO networks with varied $\bar{d}$ = [4, 12, 20]. **(b)** nPSO networks have been generated with parameters N = 100, $\bar{d}$ = [4, 12, 20], T = [0.1, 0.5, 0.9], γ = [2, 2.5, 3] and C = 4. For each network, we computed the TSP for all nonadjacent node pairs and then assessed the proportion of pairs characterized by each TSP length from 2 to 8 (the maximum TSP length among the networks generated). The results of such TSP proportions are reported in 3 heatmaps corresponding to $\bar{d}$ = [4, 12, 20], every row represents a different combination of parameters T and γ and every column a TSP length.

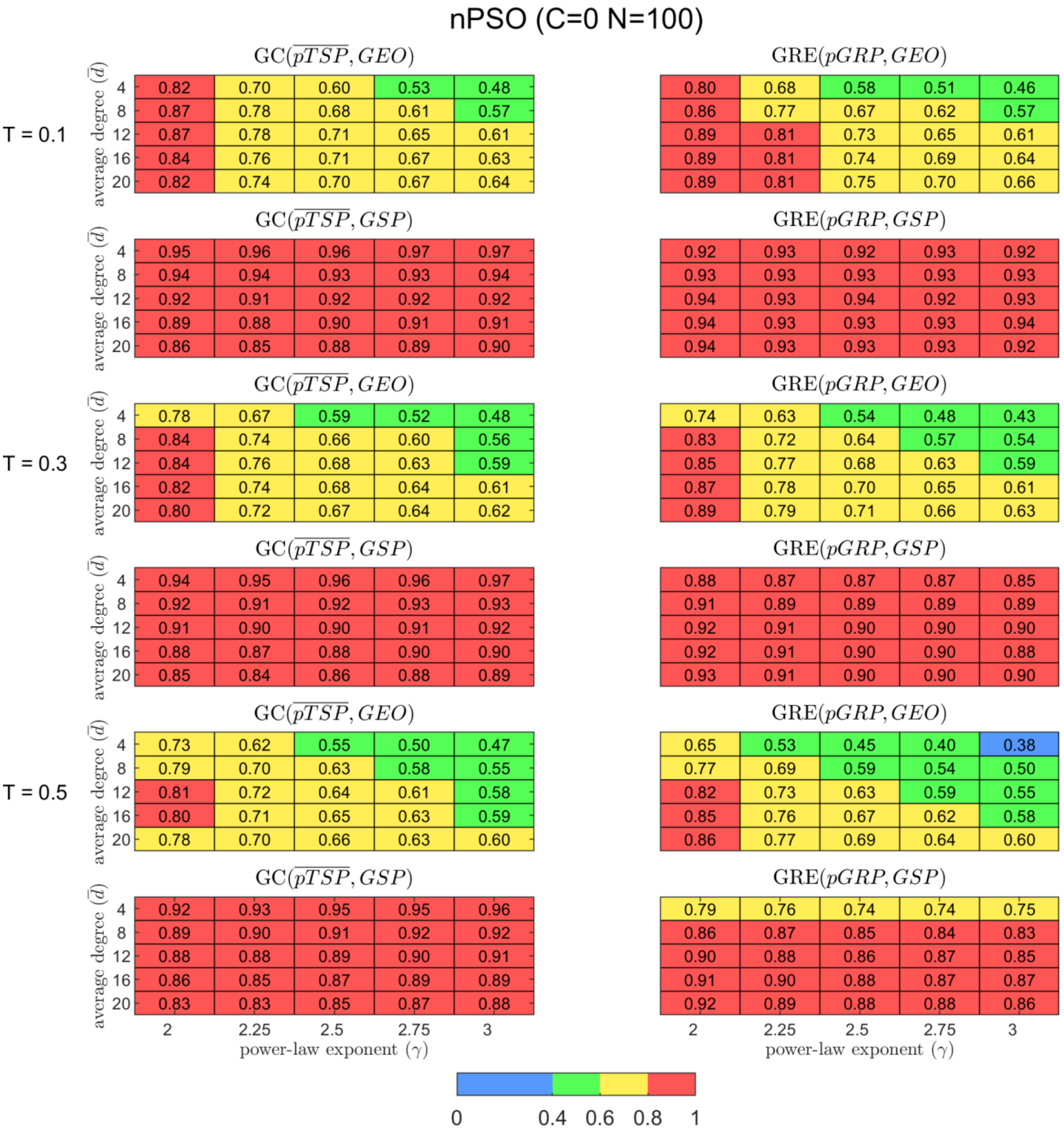

Suppl. Figure 2. GC and GRE evaluation on nPSO networks (C = 0, N = 100).

nPSO networks have been generated with parameters N = 100, $\bar{d}$ = [4, 8, 12, 16, 20], T = [0.1, 0.3, 0.5], γ = [2, 2.25, 2.5, 2.75, 3] and C = 0. For each combination of parameters, 10 networks have been generated. For each network we have computed: GC($\overline{pTSP}$, GEO), GRE(pGRP, GEO), GC($\overline{pTSP}$, GSP) and GRE(pGRP, GSP). For each value of T, indicated on the left, each heatmap reports the mean value (over 10 network realizations) of the respective network measure for each combination of $\bar{d}$ and γ in the nPSO generative model.

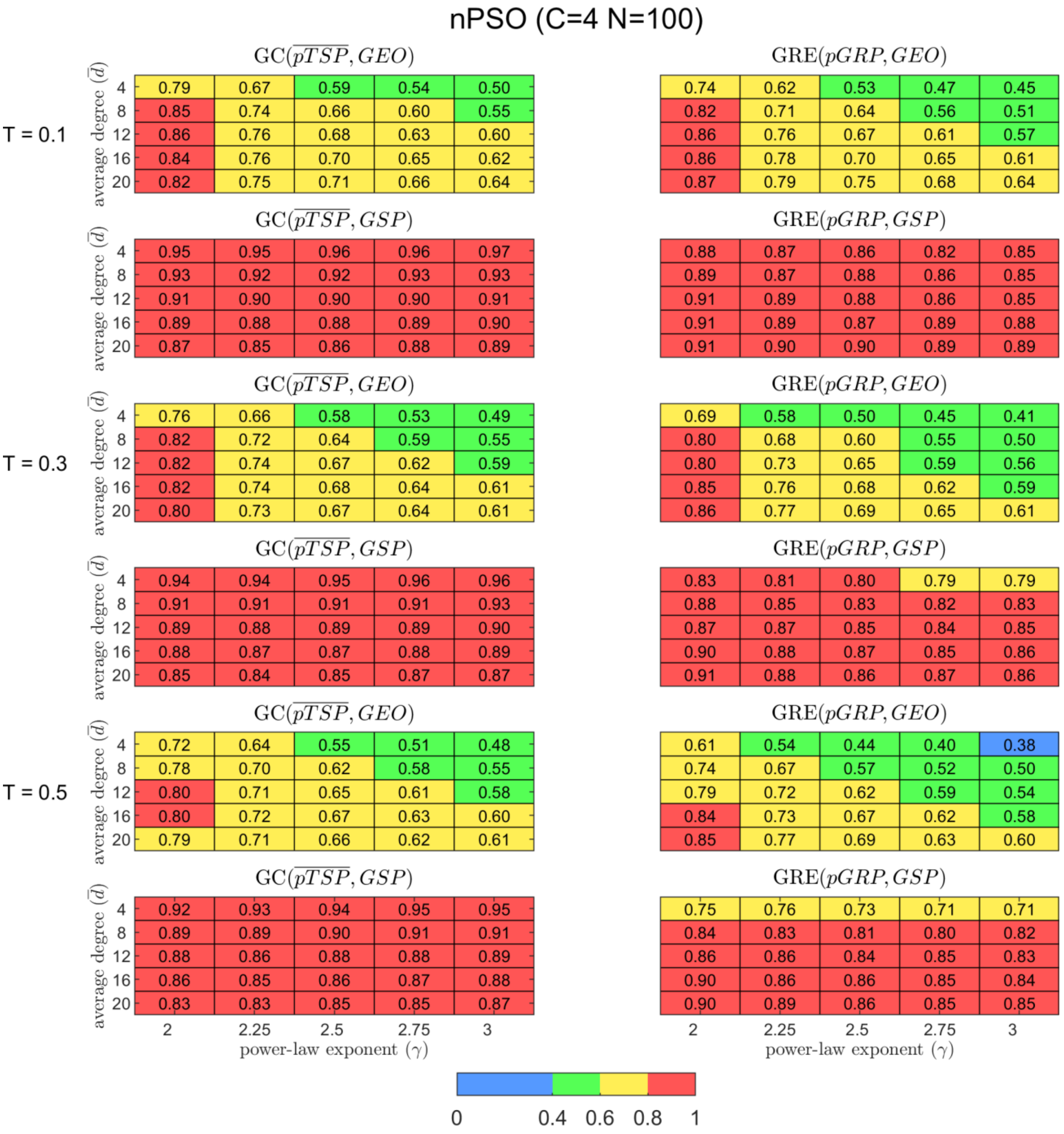

Suppl. Figure 3. GC and GRE evaluation on nPSO networks (C = 4, N = 100).

nPSO networks have been generated with parameters N = 100, $\bar{d}$ = [4, 8, 12, 16, 20], T = [0.1, 0.3, 0.5], γ = [2, 2.25, 2.5, 2.75, 3] and C = 4. For each combination of parameters, 10 networks have been generated. For each network we have computed: GC($\overline{pTSP}$, GEO), GRE(pGRP, GEO), GC($\overline{pTSP}$, GSP) and GRE(pGRP, GSP). For each value of T, indicated on the left, each heatmap reports the mean value (over 10 network realizations) of the respective network measure for each combination of $\bar{d}$ and γ in the nPSO generative model.

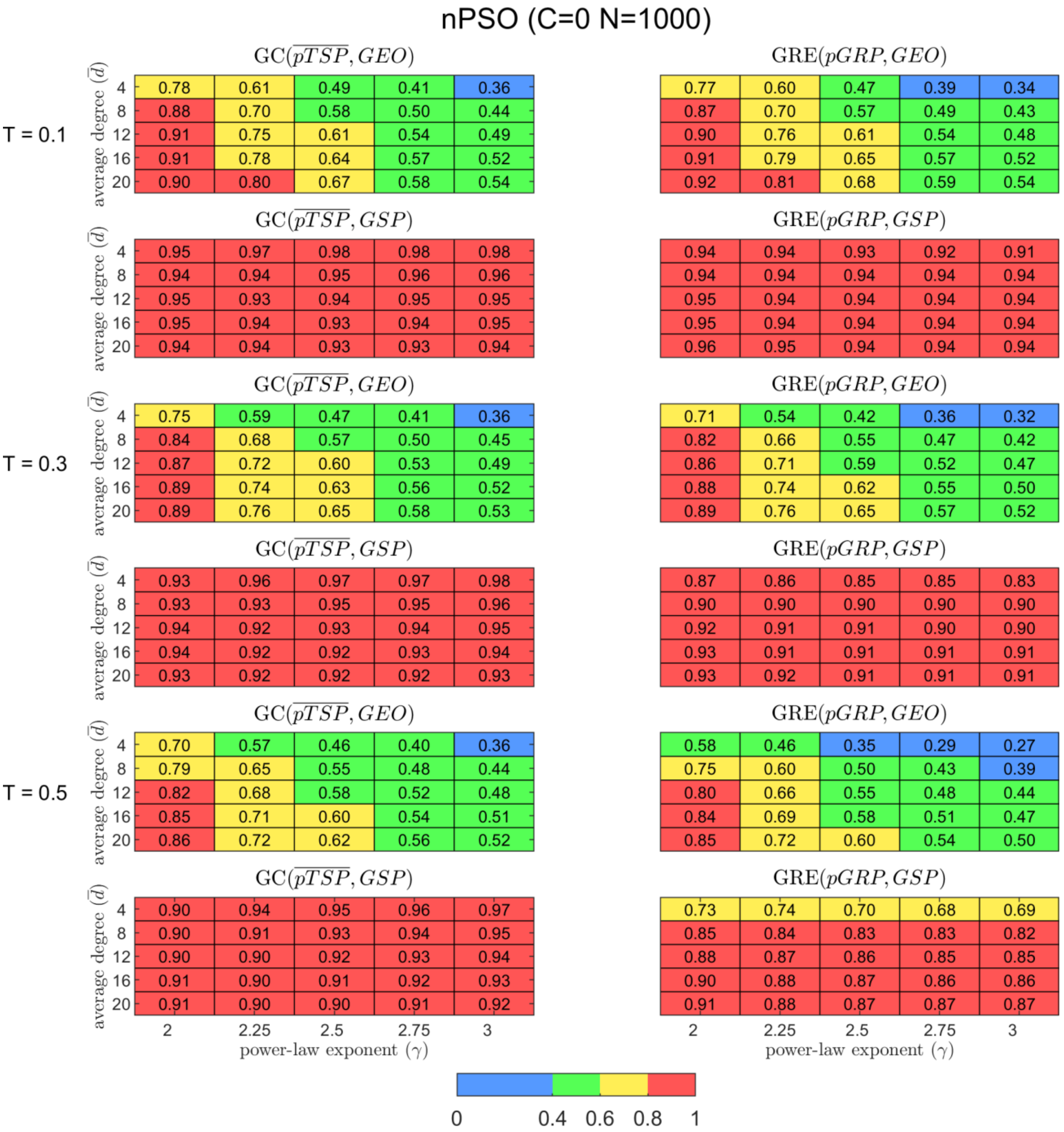

Suppl. Figure 4. GC and GRE evaluation on nPSO networks (C = 0, N = 1000).

nPSO networks have been generated with parameters N = 1000, $\bar{d}$ = [4, 8, 12, 16, 20], T = [0.1, 0.3, 0.5], γ = [2, 2.25, 2.5, 2.75, 3] and C = 0. For each combination of parameters, 10 networks have been generated. For each network we have computed: GC($\overline{pTSP}$, GEO), GRE(pGRP, GEO), GC($\overline{pTSP}$, GSP) and GRE(pGRP, GSP). For each value of T, indicated on the left, each heatmap reports the mean value (over 10 network realizations) of the respective network measure for each combination of $\bar{d}$ and γ in the nPSO generative model.

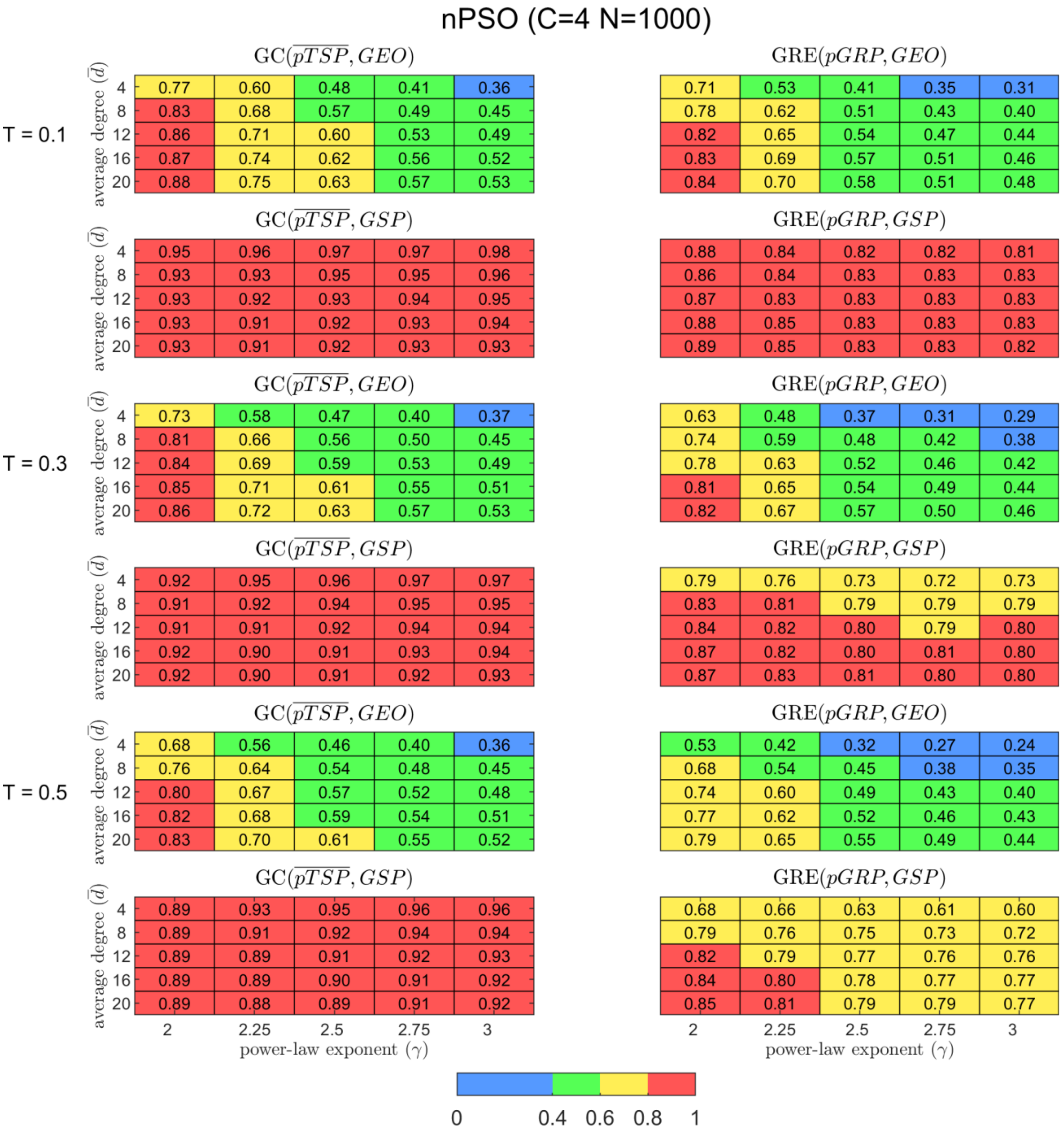

Suppl. Figure 5. GC and GRE evaluation on nPSO networks (C = 4, N = 1000).

nPSO networks have been generated with parameters N = 1000, $\bar{d}$ = [4, 8, 12, 16, 20], T = [0.1, 0.3, 0.5], γ = [2, 2.25, 2.5, 2.75, 3] and C = 4. For each combination of parameters, 10 networks have been generated. For each network we have computed: GC($\overline{pTSP}$, GEO), GRE(pGRP, GEO), GC($\overline{pTSP}$, GSP) and GRE(pGRP, GSP). For each value of T, indicated on the left, each heatmap reports the mean value (over 10 network realizations) of the respective network measure for each combination of $\bar{d}$ and γ in the nPSO generative model.

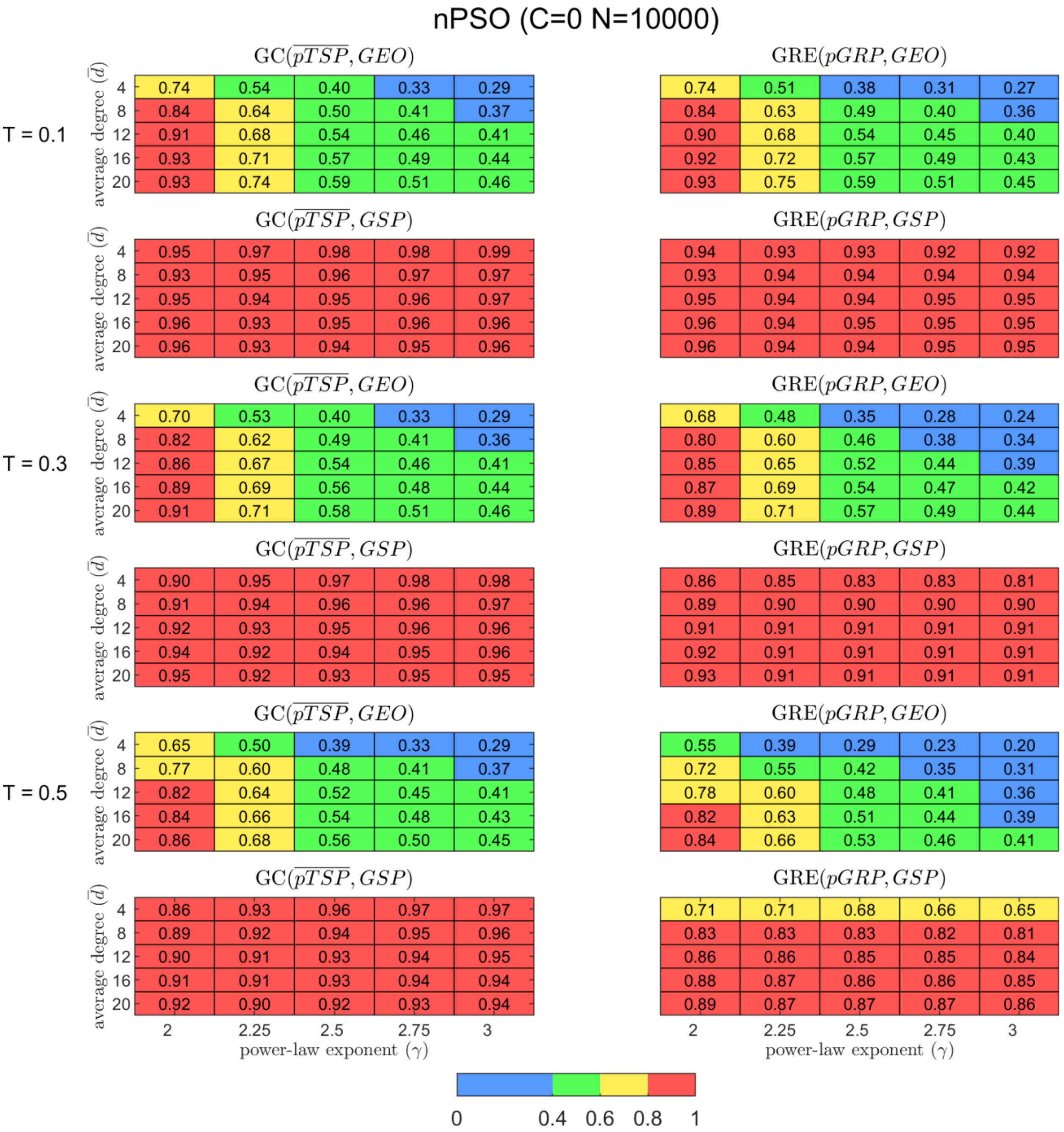

Suppl. Figure 6. GC and GRE evaluation on nPSO networks (C = 0, N = 10000).

nPSO networks have been generated with parameters N = 10000, $\bar{d}$ = [4, 8, 12, 16, 20], T = [0.1, 0.3, 0.5], γ = [2, 2.25, 2.5, 2.75, 3] and C = 0. For each combination of parameters, 10 networks have been generated. For each network we have computed: GC($\overline{pTSP}$, GEO), GRE(pGRP, GEO), GC($\overline{pTSP}$, GSP) and GRE(pGRP, GSP). For each value of T, indicated on the left, each heatmap reports the mean value (over 10 network realizations) of the respective network measure for each combination of $\bar{d}$ and γ in the nPSO generative model.

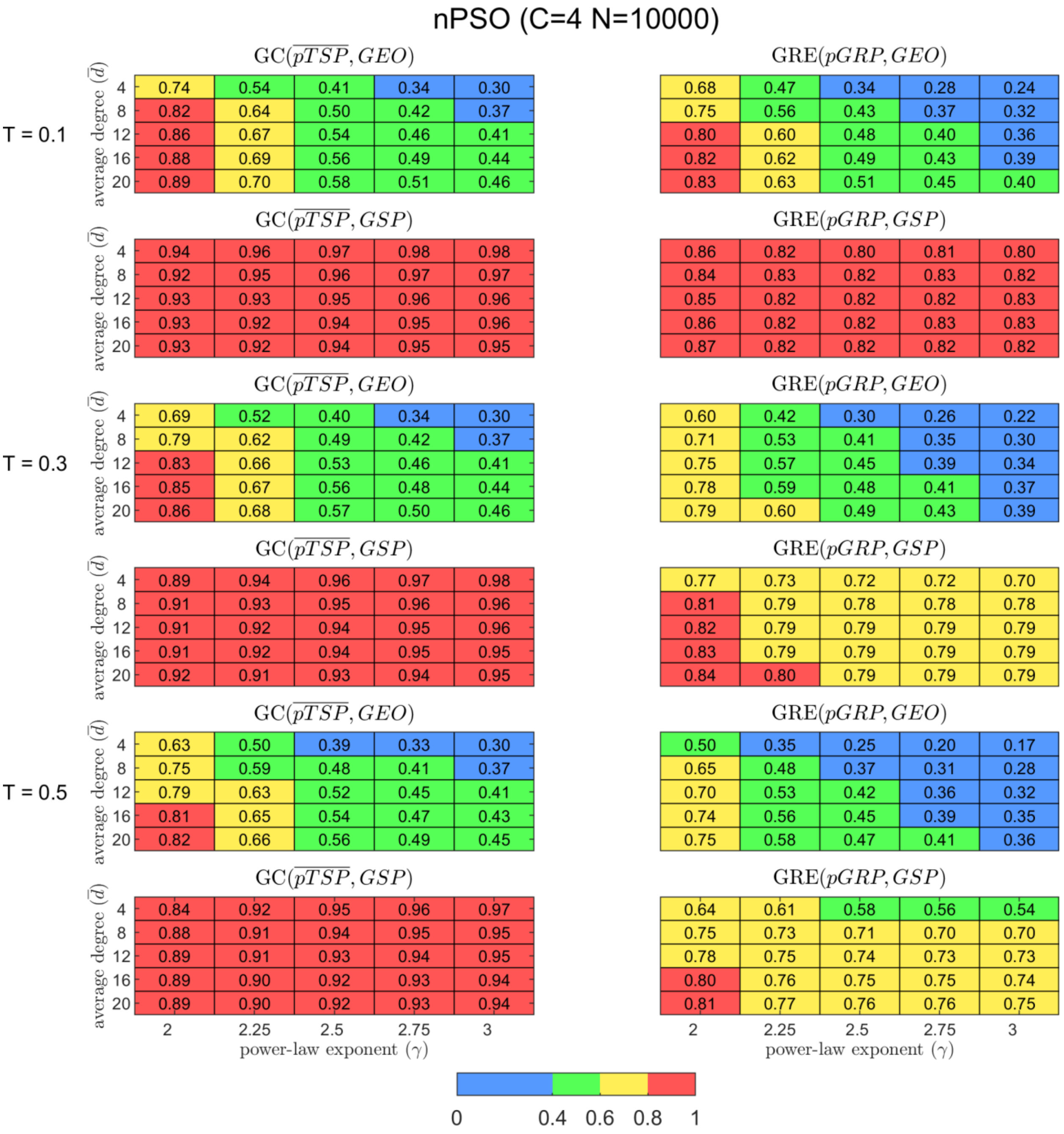

Suppl. Figure 7. GC and GRE evaluation on nPSO networks (C = 4, N = 10000).

nPSO networks have been generated with parameters N = 10000, $\bar{d}$ = [4, 8, 12, 16, 20], T = [0.1, 0.3, 0.5], γ = [2, 2.25, 2.5, 2.75, 3] and C = 4. For each combination of parameters, 10 networks have been generated. For each network we have computed: GC($\overline{pTSP}$, GEO), GRE(pGRP, GEO), GC($\overline{pTSP}$, GSP) and GRE(pGRP, GSP). For each value of T, indicated on the left, each heatmap reports the mean value (over 10 network realizations) of the respective network measure for each combination of $\bar{d}$ and γ in the nPSO generative model.
